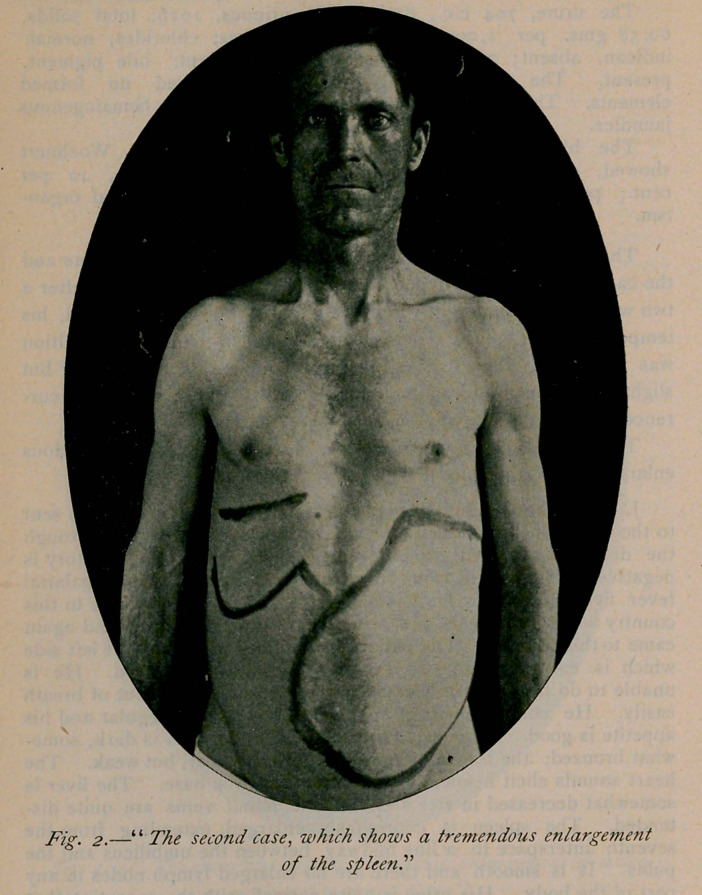# Malaria Representing Three Varieties Observed in Buffalo

**Published:** 1901-07

**Authors:** Julius Ullman

**Affiliations:** Buffalo, N. Y.; Instructor of clinical medicine, Med. Dept. University of Buffalo; attending physician German Hospital; attending physician, University of Buffalo Dispensary


### MALARIA REPRESENTING THREE VARIETIES OBSERVED IN BUFFALO.

By JULIUS ULLMAN, M. D., Buffalo, N. Y.,

Instructor of clinical medicine, Med. Dept. University of Buffalo; attending physician German Hospital; attending physician, University of Buffalo Dispensary.

IN A paper recently published by Lyon and Wright (Buffalo Medical Journal, November, 1900), on An enquiry into the presence of malaria in Buffalo and its environs, the authors conclusively showed that malaria is a rare disease in natives of Buffalo. Whatever cases of malaria fever have been observed of late in Buffalo were imported, and more especially noted in those soldiers returning from the tropics, particularly from Cuba. The cases usually noted belong to the tertian or quotidian types, but occasionally the more pernicious form is seen—namely, the estivo-autumnal variety.

It is now agreed (Malaria, Encyclopedia Medica, Vol. VII.) that there are various species of malarial organisms which will only reproduce their kind, each of which is capable of reproducing the respective varieties alluded. It is also shown that the organisms have two cycles of reproduction, asexual as seen in the infection of the human species, and an extra- or asexual, having the mosquito as the intermediary host. The crescent bodies, those organisms first described by Leveran, are seen in the estivo-autumnal fever.

The following illustrative case is reported, showing the malarial cachexia, and the enormous hepatic and splenic enlargement of a case of estivo-autumnal variety of malarial fever.

P. J. B., aged 30; Irish and a teamster; was admitted to the German Hospital, May 9, 1901.

*Family history.*—Father and mother are both living and well; two sisters and six brothers are alive. There is no obtainable history of tuberculosis, syphilis, diabetis mellitus or rheumatism, nor have any relatives ever died of cancer.

*Past history.*—Patient has had measles and diphtheria; eight years ago had typhoid fever. Denies having had any specific disease. Has used alcohol freely.

*Present history.*—In 1898, patient went to Cuba and was engaged with the army, his duties calling him to different parts of Cuba. He remained in Cuba until April, 1901. During the first year and a half, the patient enjoyed good health, but after this period, while in Porto Principe and Santiago, he had chills and fever, for which he took large quantities of quinine. At the time of his illness, he became suddenly dizzy and vomited freely. He had no appetite, was chilly and had a rise in temperature; the bowels were constipated and he suffered severe headaches. This attack lasted four days and following it, he had illnesses of the same nature, but of more or less severe character. An aura was present in the majority of attacks and consisted in a burning or tickling sensation in the feet, severe thirst and headache and general feeling of tenderness, particularly in the region of the kidney—so-called by patient, but more probably the spleen. Frequent micturition, particularly at night, was a frequent symptom. Nausea and vomiting were not infrequent. When the patient was indoors or in the shade, the attacks occurred less frequently; while the heat and sun seemed to precipitate a paroxysm. The paroxysms were most prone to occur after dinner, at 2 or 3 o'clock p. m., and abate when the coolness of the evening came on.

When in Cuba the patient took very large doses of quinine, often sufficient to produce a temporary deafness, with but slight effects upon the disease. His condition improved slightly on his leaving Cuba, but mild paroxysms still continued.

*Status presens.*—The patient is of good bony frame, but is anemic, and evidently has lost much weight and strength. His face is sallow, skin dry, and instinctively gives to the examiner the picture of a malarial cachexia. The pulse was regular and well sustained, but the capillary circulation is poor. The patient has no appetite and sometimes vomits.

*Fig. 1.—" The enormous hepatic and splenic enlargement of estivo-autumnal malarial fever."*

Inspection of the abdomen by means of the Litten shadow test, the lower border of the liver may easily be seen on a line of the umbilicus and in deep inspiration descending below it. On percussion, the hepatic and splenic dulness is increased, and on palpation is shown, as illustrated. The respiratory apparatus gives a negative finding. The heart sounds reveal hemic bruits over the base and a bruit diable is elicited over the jugular vein of the wall.

The urine, 704 c.c., dark, acid; urinous, 1026; total solids, 60.58 gms. per 1,000 c.c.; urea, 18.3 gms; chlorides, normal; indican, absent; albumin, absent; sugar, absent; bile pigment, present. The microscopical examination, showed no formed elements. The bile pigment was evidently due to the hematogenous jaundice.

The blood which was examined by Dr. A. E. Woehnert showed, reds, 2,500,000; whites, 3,200. Hemoglobin, 40 per cent.; poiklocytosis, and the crescent form of the malarial organism.

The patient was placed on large doses of arsenic with quinine and the cacydelate of soda hypodermatically and left the hospital after a two weeks' sojourn, slightly improved. His chills were checked; his temperature and pulse remained normal and his general condition was improved. The liver and splenic dulness were, however but slightly influenced and, undoubtedly, he will soon again have a recurrence of his malarial symptoms.

The second case, which shows, as illustrated, a tremendous enlargement of the spleen, is of unusual interest:

J. La T., aged 49; a laborer and a native of Italy. He was sent to the wards of the German Hospital by Dr. M. Hartwig, and through the doctor, I obtained the following history. The family history is negative. The patient came from Sicily. In Sicily he had malarial fever five years ago. He has had chills and fever and came to this country some three years ago, when he returned to Italy and again came to this country. The patient has noticed a mass in the left side which is easily palpable and corresponds to the spleen. He is unable to do heavy work because of weakness, and gets out of breath easily. He complains of no pain; his bowels are regular and his appetite is good. Patient is fairly nourished; his skin is dark, somewhat bronzed; the tongue is normal; pulse, regular, but weak. The heart sounds elicit hemic, systolic bruits over the base. The liver is somewhat decreased in size and the abdominal veins are quite distended. The spleen is enormously enlarged, extending from the seventh interspace to a line midway between the umbilicus and the pubis. It is smooth and there are no enlarged lymph nodes in any part of the body. His urine is quite normal, with the exception that a trace of bile was once noted.

During his observation in the hospital no hemorrhages were noted nor was there any rise of temperature. For the purpose of diagnosis Dr. A. E. Woehnert examined the blood: reds, 3,532,000; whites, 4,400; hemoglobin, 40 per cent. There are many microcytes, a moderate number of macrocytes, poiklocytes. A differential count of the whites give: polymorphous leucocytes, 72 per cent.; small lymphocytes, 21 per cent.; large lymphocytes, 6 per cent.; eosino-

*Fig. 2.—"The second case, which shows a tremendous enlargement of the spleen."*

philes, 1 per cent.; total, 100 per cent. There are no plasmodia malaria and no increase of fibrin.

This accords in many respects, especially in the blood examination to splenic anemia, described in Allbut's System of Medicine, p. 539, as "a form of profound anemia, progressive in character, ending fatally; generally of no long duration, associated with great enlargement of the spleen, but without leucocytosia or enlarged glands. It has also been called splenic cachexia, splenic pseudoleukemia, lymph adenoma splenicum and splenomegaly primitive." This case agrees with this description in (1) the anemia; (2) the enlarged spleen; (3) the absence of leucocytosis and enlarged glands elsewhere, but there seems to be a causal relation from his history to malaria, and so we must further regard the great enlargement of the spleen to chronic malaria, and the small size of the liver to a simple or marantic atrophy, often seen in chronic malarial cases. The blood count, though the malarial organism has not been found and which is not so unusual in the chronic cachectic cases, is a picture of a chloro-anemia, due to the chronic malarial intoxication.

Our case, then, presents well the clinical and blood picture of a splenic anemia with the additional factor of malarial origin.

A third case, which shows a typical attack of tertian malarial fever, and which is of especial interest to us from the fact that the patient has never previously had an attack of malaria, and has lived in Buffalo continuously for a year, is as follows.

B.F., aged 21; German; came to the medical ward of the German Hospital, June 5, 1901. The family history is negative. Patient has had the exanthemata, and since childhood has been very well. He lived in Berlin, Germany, 17 years, and then accepted a position as fireman on an ocean steamer. He traveled from America to Hamburg and back as fireman for one year. Following this he traveled between Australia, Hamburg, China and Brazil and during the entire career was only troubled with seasickness. Upon entrance to hospital patient had temperature, 103; pulse, 130. He complained of chills, fever, nausea and vomiting. He was constipated; both liver and spleen were enlarged, the latter being palpable; the tongue was coated and furred.

To our astonishment the temperature was normal on the following day, June 6, when at 10 a.m. of the 7th, being the same time as rise of June 5, he again had a paroxysm; a calm on 8th and a paroxysm at same hour on 9th, so also on the 11th.

The blood was examined and the plasmodium malaria found, when quinine was given, since which time his temperature has remained normal and no further paroxysms have occurred

400 FRANKLIN STREET.

## Figures and Tables

**Fig. I. f1:**
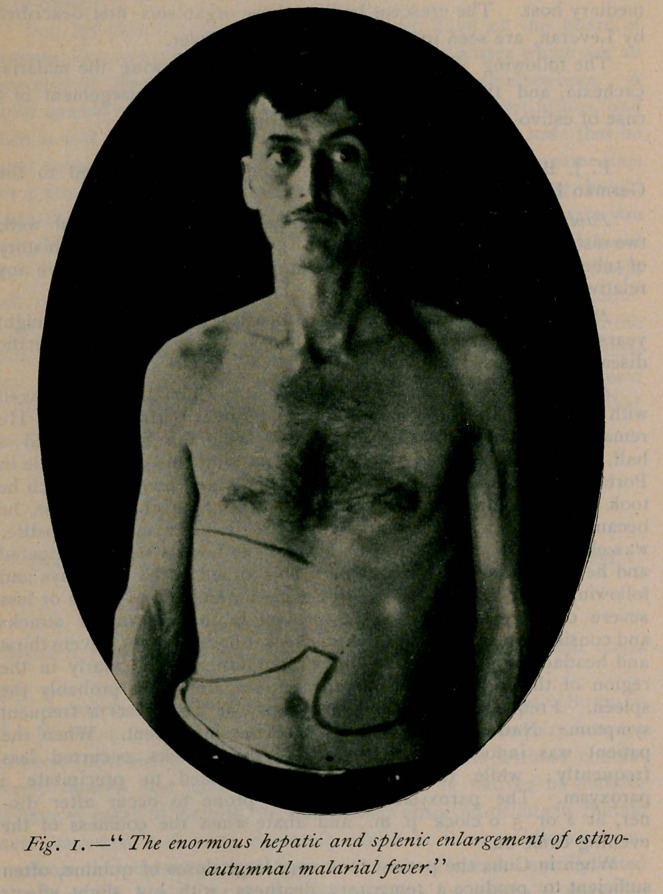


**Fig. 2. f2:**